# A pilot study on dasatinib in patients with Waldenström macroglobulinemia progressing on ibrutinib

**DOI:** 10.1002/jha2.493

**Published:** 2022-06-07

**Authors:** Jorge J. Castillo, Shayna Sarosiek, Catherine A. Flynn, Carly Leventoff, Megan Little, Timothy White, Kirsten Meid, Steven P. Treon

**Affiliations:** ^1^ Bing Center for Waldenström Macroglobulinemia Dana‐Farber Cancer Institute Boston Massachusetts USA; ^2^ Harvard Medical School Boston Massachusetts USA

**Keywords:** clinical trial, dasatinib, HCK, Waldenström macroglobulinemia

## Abstract

The hematopoietic cell kinase (HCK) regulates BTK activation and represents a potential therapeutic target in Waldenstrom macroglobulinemia (WM). We investigated dasatinib, a potent HCK inhibitor, in patients with WM progressing on ibrutinib. Study treatment consisted of dasatinib administered at 100 mg by mouth once daily in four‐week cycles for up to 24 cycles. This study was registered under ClinicalTrials.Gov ID NCT04115059. Three participants were enrolled and received at least one cycle of dasatinib. The best response was stable disease. Two patients received 5 months and one patient received 1 month of therapy. The dose of dasatinib was decreased in one participant due to volume overload. Based on the lack of responses observed, the study was terminated. Dasatinib might not be effective in patients with WM progressing on ibrutinib.

## 1 INTRODUCTION

The prevalence of the somatic MYD88 L265P mutation is over 90% in patients with Waldenstrom macroglobulinemia (WM), and the Bruton tyrosine kinase (BTK) pathway plays an essential role in the signaling of mutated MYD88 providing survival advantage and proapoptotic mechanisms to malignant WM cells [1]. The covalent BTK inhibitors ibrutinib (with and without rituximab) and zanubrutinib are the only drugs approved by the United States Food & Drug Administration (FDA) for the treatment of patients with WM [2, 3, 4]. The acquisition of BTK mutations (i.e., BTK C481S) induces resistance to covalent BTK inhibitors in WM cells through a paracrine mechanism [5]. However, the best treatment option for patients whose disease progress on BTK inhibitors is not standardized [6].

The hematopoietic cell kinase (HCK) is aberrantly expressed in WM cells and is also a target of ibrutinib [7]. Furthermore, HCK regulates BTK activation, mediates pro‐survival signaling in MYD88‐mutated WM cells, and represents a potential therapeutic target [8, 9]. Dasatinib, a BCR‐ABL inhibitor FDA‐approved for the treatment of patients with chronic myelogenous leukemia, is also a potent HCK inhibitor [10]. Therefore, we designed a prospective pilot study to investigate the safety and efficacy of dasatinib in patients with WM who were progressing on ibrutinib.

## 2 METHODS

This is a prospective, single center, single arm, pilot study. Selected inclusion criteria were age ≥18 years, good performance status (Eastern Cooperative Oncology Group ≥2), clinicopathological diagnosis of WM based on the 2nd International Workshop for WM (IWWM‐2) [11], presence of the *MYD88 L265P* mutation, disease progression based on IWWM‐6 [12], symptoms associated with disease progression [13], and good organ and marrow function (i.e., neutrophil count ≥500/µl, platelet count ≥50,000/µl, hemoglobin level ≥7 g/dl, estimated glomerular filtration rate ≥30 ml/min, and aminotransferase levels ≤2.5 times the upper limit of normal). Selected exclusion criteria were prior exposure to BCR‐ABL inhibitors, known central nervous system involvement, active HIV, hepatitis B or hepatitis C infection, and QTc interval > 450 ms on pre‐entry electrocardiogram. Study treatment consisted of dasatinib administered at a dose of 100 mg by mouth once daily in four‐week cycles for up to 24 cycles. Dose reductions were allowed for toxicity. Participants who received at least one dose of dasatinib were evaluable for safety, and participants who received at least one cycle were evaluable for response. Routine laboratory studies, bone marrow aspirate and biopsy for *MYD88*, *CXCR4*, *BTK* and *PLCγ2* mutations analysis, and computed tomography scans of the chest, abdomen and pelvis with intravenous contrast were performed at baseline. Responses were assessed using criteria from IWWM‐6 [12]. This pilot study aimed at enrolling six patients. Funding was provided by a Dunkin Translational Breakthrough Grant. A limited drug supply for six participants was provided by Bristol‐Myers Squibb, the manufacturer of dasatinib, for this pilot study. Given the study drug supply limitations, this study was meant to be hypothesis‐generating rather than hypothesis‐testing. This study was registered under ClinicalTrials.Gov ID NCT04115059.

## 3 RESULTS

Three participants were enrolled in this study. Participant 1 was a 73‐year‐old man who had 10 previous lines of therapy, which included bendamustine, cyclophosphamide, doxorubicin, rituximab, alemtuzumab, everolimus, bortezomib, thalidomide and ibrutinib, and met criteria to treat based on fatigue, shortness of breath, and symptomatic extramedullary disease (soft tissue mass in the oral cavity). Baseline hemoglobin was 9.6 g/dl, with normal white blood cell (WBC) and platelet counts, and serum IgM level was 710 mg/dl. There was no lymphadenopathy or organomegaly at baseline. The malignant cells harbored *BTK C481S* but no *CXCR4* mutations. Serum IgM level peaked at cycle 3 at 1809 mg/dl, likely an IgM rebound off ibrutinib, to later decrease to 1129 mg/dl by cycle 5. The best response was stable disease. Dasatinib dose was reduced to 70 mg and then to 50 mg once daily because of volume overload and heart failure. The patient progressed symptomatically, and treatment was stopped after 5 months of dasatinib exposure. Four weeks later, the patient was admitted to the hospital with fever but without hemodynamic compromise. He was found unresponsive in his hospital room and resuscitation efforts were futile. The participant died 6 months from dasatinib exposure. Participant 2 was a 46‐year‐old woman who had one previous line of therapy (ibrutinib) and met criteria to treat based on symptomatic anemia. Baseline hemoglobin level was 7.3 g/dl, platelet count was 98 K/µl, WBC count was 3.2 K/µl, and serum IgM level was 6,516 mg/dl. Imaging revealed no lymphadenopathy but there was splenomegaly. Malignant cells harbored a *PLCγ2* mutation but there were no *CXCR4* mutations. The patient withdrew consent after one cycle of therapy because of lack of response. At the end of the treatment, serum IgM was 6,750 mg/dl. The best response to dasatinib was stable disease. The participant went on to receive bendamustine and rituximab. Participant 3 was a 75‐year‐old woman who had two previous lines of therapy, which included bortezomib, dexamethasone, rituximab, and ibrutinib, and met criteria due to symptoms of hyperviscosity. The baseline hemoglobin level was 14.9 g/dl, WBC count was 5.8 K/µl, platelet count was 141 K/µl, and serum IgM level was 2,642 mg/dl (after plasmapheresis). CT scans revealed lymphadenopathy but no splenomegaly. The malignant cells did not harbor *CXCR4* mutations, and the *BTK* or *PLCγ2* mutational status was unknown. The best response to dasatinib was stable disease, and study treatment was stopped after 5 months because of disease progression with serum IgM level at the end of therapy of 5,489 mg/dl. The participant went on to receive venetoclax.

Based on the lack of responses observed in this pilot study after enrolling three participants, we decided to terminate the study.

## 4 DISCUSSION

With the FDA approval of covalent BTK inhibitors, targeted agents have taken center stage in the treatment of patients with WM. We performed the present pilot study with dasatinib encouraged by the findings of our preclinical studies. However, dasatinib might not be effective at inducing responses in patients with WM who are progressing on ibrutinib. There are promising emerging treatment options in patients who are actively progressing on covalent BTK inhibitors, however. The BCL2 antagonist venetoclax was safe and effective in a phase II study in patients with previously treated WM, in which half of the participants were previously exposed to covalent BTK inhibitors, with an overall response rate of 83% and a median progression‐free survival of 30 months [14]. Venetoclax was recently added to the National Comprehensive Cancer Network (NCCN) guidelines as a treatment option for previously treated patients with WM. Additionally, the noncovalent BTK inhibitor pirtobrutinib have shown early efficacy with a distinct adverse event profile in a phase I/II study in patients with previously treated B‐cell malignancies [15]. Pirtobrutinib induced a response in six of the eight patients with WM who were progressing on a covalent BTK inhibitor, without evidence of arrhythmia, a well‐known adverse event associated with covalent BTK inhibitors. Recently, mechanisms of resistance to noncovalent BTK inhibitors have been identified, including the development of mutations in *BTK* (i.e., *V416L*, *A428D*, *M437R*, *T474I*, and *L528W*) [16]. Some of these mutations are predicted to induce resistance to noncovalent BTK inhibitors. We recently published our preclinical experience with the dual HCK/BTK inhibitor KIN‐8194, which showed activity against WM cell lines with *BTK C481S* mutations, with enhanced activity in xenografted mice with the addition of venetoclax [17].

Given the small‐sample size of our pilot study, strong conclusions on the efficacy of dasatinib in WM progressing on ibrutinib cannot be reached. Although our study was not testing a specific hypothesis, we would have hoped for a response rate of at least 50%. After observing stable disease in three participants, we felt attaining such response rate would be unlikely. Dasatinib might not be effective in patients with WM progressing on ibrutinib. Therefore, other approaches to treat patients with WM progressing on covalent BTK inhibitors should be sought.

## CONFLICT OF INTEREST

Jorge J. Castillo received research funds and/or honoraria from Abbvie, AstraZeneca, Beigene, Casma, Cellectar, Janssen, Pharmacyclics, Polyneuron, Roche and TG Therapeutics. Shayna Sarosiek received research funds and/or honoraria Acrotech Biopharma, ADC Therapeutics and BeiGene from Steven P. Treon received research funds and/or honoraria from Abbvie, Beigene, BMS, Pharmacyclics and X4.

## AUTHORS CONTRIBUTION

Jorge J. Castillo and Steven P. Treon designed the study. Carly Leventoff, ML, Kirsten Meid and Timothy White provided administrative support. Jorge J. Castillo and Shayna Sarosiek provided patients. Jorge J. Castillo, Shayna Sarosiek, Kirsten Meid and Steven P. Treon analyzed the data. Jorge J. Castillo drafted the initial manuscript. All the authors critically reviewed the manuscript and approved the final version.

## ETHICS STATEMENT

The Dana‐Farber Cancer Institute Institutional Review Board approved this study (DFCI 19‐305). All the patients provided written consent before participation in this study.

## Data Availability

Data can be requested by email to Dr. Jorge J. Castillo at jorgej_castillo@dfci.harvard.edu.
